# “Sex and Ceruloplasmin Modulate the Response to Copper ...”

**DOI:** 10.1289/ehp.113-a226a

**Published:** 2005-04

**Authors:** Tony Tweedale

**Affiliations:** Montana-CHEER (Coalition for Health, Environmental & Economic Rights), Missoula, Montana, E-mail: ttweed@wildrockies.org

It is past time for *EHP* to stop accepting papers whose funding disclosure makes it obvious there is a conflict, yet “The authors declare they have no competing financial interests.” Méndez et al. (2004) stated that “This investigation was funded by the International Copper Association [ICA] in the form of an unrestricted research grant.” I fail to see that an unrestricted grant eliminates a conflict of interest. From the article’s introduction, it is obvious that this research team has done a lot of copper toxicity work with this money from the ICA, but the conflict of interest would exist even if only the work reported in this article was funded by the ICA.

It is interesting, however, that the ICA is funding human experimentation. The pesticide industry’s push to allow human experimentation in the toxicity tests to register their pesticides in the United States is driven by their resulting ability to drop the 10-fold interspecies safety factor in allowable exposure levels. Lockwood (2004) found all six human pesticide studies reviewed rife with financial conflict of interests. Think of the risks created to all toxicity testing when the most reputable general toxicology journal in the world, *EHP*, endorses human subjects for toxicity testing in very risky situations.

Many of us tolerate animal testing because we hope that eventually the current massive risk of toxic agents will be acknowledged.

It may seem that this work on Cu entailed little risk, as the authors claim in the opening of their discussion:

… Liver aminotranferases were evaluated to satisfy ethical considerations. We detected no responses that may represent toxic effects of the Cu dose used.

First, the authors acknowledged that there are large data gaps on the toxicity of Cu at many doses. Critically, this demonstrable truth makes their statement about detecting no toxic responses false. Obviously they were not looking at many toxic end points—especially chronic effects. Also, they stopped looking for any effects after a very short period (82 subjects ingested 10 mg/kg/day Cu for 2 months).

The stated tolerable daily intake (TDI) in the introduction (Méndez et al. 2004) is unclear (and unattributed), but it appears to range from 0.9 to 10 mg/kg/day. The experimental dose chosen for this study was 10 mg/kg/day, and was justified by the authors as being a dose safe for 97.5% of humans. TDIs are typically derived from industry junk science (unpublishable in independent journals) and contain massive data gaps. However, even if we assume the claimed TDI is validated, the authors are admiting that their chosen experimental dose was above the “safe” level for about two of their subjects.

At the end of the discussion the authors admit that the Cu-induced enzyme changes they looked at are altered by hepatic diseases; Méndez et al. (2004) then state that their results can be used to monitor adverse liver effects.

The effect of the combination of risky dosing with acknowledged toxicity data gaps is stunning. In summary, I am disappointed that *EHP* ’s manuscript reviewers and editors allowed such dangerous (unethical) statements and objective inconsistencies; I fear that a human toxicity experiment—in *EHP* of all places—has created a terrible precedent; and I am frustrated that authors and *EHP* continue to misstate obvious conflicts of interest. I look forward to discussion and solutions.
